# Recursive expectations approach in policymaking

**DOI:** 10.1007/s42495-023-00108-w

**Published:** 2023-05-09

**Authors:** Keiichiro Kobayashi

**Affiliations:** Keio University, Tokyo, Japan

**Keywords:** Recursive expectations, Nonperforming loans, Unconventional monetary policy, COVID-19, PCR testing, E50, G01, H30, I00

## Abstract

The primary contribution of modern economics to policy analysis may be the recognition of the crucial role of expectations in policy intervention. The essence of expectations is to think about others’ thinking. We argue that policymakers need *recursive thinking*, that is, the ability to think about thinking and review three policy episodes related to the lack of recursive thinking. We see that the disciplinary divide or the limited scope of recursive thinking on the side of policymakers can cause huge damage to social welfare in times of crisis. Finally, we consider two examples of future agendas in the recursive approach.

## Introduction

### Recursive expectations’ equilibrium

In standard economics, we use the notion of rational expectations to argue that the effectiveness of economic policy may be much less than the level that was supposed in Old Keynesian economics. The pioneering work of this line of argument is the Lucas critique [16], and the most prominent example of the critique is the argument of Ricardian equivalence that claims no effectiveness of fiscal policy [3].

The notion of rational expectations consists of two components. The first component is *perfect rationality*, or complete information and logical and scientific thinking, which is the rationality that we use as a non-technical word in daily life. Complete information means that the agent knows everything she/he needs, and logical and scientific thinking means that the agent can make accurate inferences in a scientific manner. This assumption of perfect rationality has been criticized over the years for being too strong as an assumption for the basis of human behavior. There is, however, the second component in the notion of rational expectations, which is the core of the notion and is much more indispensable than perfect rationality. That is, *recursive thinking*, or the ability to think about thinking. I agree with [5], who says, “the ability to think about thinking may be the critical attribute that distinguishes us from all other species.” We can define the notion of *recursive expectations* as the expectations held by people who have recursive thinking, that is, the ability to think about others’ thinking. Recursive expectations are a broader concept than rational expectations, as recursive expectations necessitate only recursive thinking, whereas rational expectations are recursive expectations plus perfect rationality. When people have the ability to think about thinking, I expect what you expect, and you expect what I expect. Thus, I expect what you expect. You expect that I expect what you expect. I expect that you expect that I expect what you expect. This repetition continues infinitely, forming an infinite loop of expectation. We call the equilibrium of the economy, where this infinite loop of expectations holds in a consistent manner, *the recursive expectations equilibrium*.^1^

### Policymaking with recursive thinking

The essence of the Lucas critique is that the effectiveness of macroeconomic policy may be dampened in the recursive expectations equilibrium, because people have the ability to think about thinking and adjust their actions in response to others’ thinking. In particular, people are trying to outsmart policymakers’ intentions.^2^

In this paper, I would like to stress that the ability to think about thinking is most necessary for policymakers in times of crisis.^3^ The effectiveness of policy intervention and its negative side effects can be evaluated appropriately only if policymakers can consider people’s thoughts and responses. In reality, policymakers often forget to think about people seriously affected by the policy. We call this situation a lack of recursive thinking on the side of policymakers. We will see that policymakers’ lack of recursive thinking may create huge unintended damage to the economy, not to mention dampening the effectiveness of the policy.

## Policy failures due to lack of recursive thinking

Lucas [16] criticism is that Keynesian economics completely lacks recursive thinking. The policymakers at that time who adopted the Keynesian framework assumed that people mechanically reacted to the policy intervention, as prespecified by the consumption function or the investment function. They implicitly regarded an economy that consists of uncountable people as an unintelligent entity that responds mechanically to their policy, just like a machine or a brainless amoeba that withdraws its body in response to a stimulus.

Today, policymakers have become more serious about trying to think about people’s thinking. The problem today is *who’s thinking* that policymakers care about. The scope of the people that policymakers care about is limited according to their jurisdiction in normal times. Policymakers consider the cost and effect of their policies in recursive thinking on the premise that people in their scope also have the ability to think about thinking. However, they may simply cease considering the people outside of their scope or simply assume that people outside of their scope will not change their actions in response to the policy interventions.

### How is the scope of recursive thinking determined?

We may call this policymakers’ attitude the lack of recursive thinking, or more precisely, the *limited scope* of recursive thinking. The limited scope could also be rephrased as a jurisdictional divide or disciplinary divide when the nature of the policy is highly professional. The typical disciplinary divide seen below is the divide in public health policy during the COVID-19 pandemic. Policymakers care about COVID-19 patients and medical doctors, and their attention is hardly focused on ordinary people and non-medical businesses that are severely affected by infection containment measures.

This study focuses on policy interventions during crisis. The limited scope of recursive thinking or the disciplinary divide can be reasonable as a way of thinking for policymakers in normal times, but it could become fatal in times of crises.

In normal times, limiting the scope of recursive thinking may be reasonable, because policy interventions are modest in normal times and affect only a small part of the economic system. According to the theory of rational inattention ([21], see [17] for a survey), we could argue that limiting the scope of recursive thinking can be an optimal choice of allocation of policymakers’ attention to maximize social welfare in normal times, given the scarcity of attention. Thus, benevolent policymakers can optimally restrict their attention to the limited scope of the people and neglect others outside their scope. They may be able to maximize social welfare in normal times by deciding on policy actions based on a limited scope. This justifies a jurisdictional or disciplinary divide.

However, in a time of crisis, necessary policy interventions could be extreme, and the scope of the people who could be affected by the policy interventions became much broader. In times of crisis, policymakers need to widen the scope of recursive thinking to appropriately evaluate the costs and effects of their policy interventions. On the contrary, if they stick to the jurisdictional or disciplinary divide and continue to limit their scope of recursive thinking, they could incur significant damage to social welfare unintentionally. Below, we observe three episodes of policy failure related to the limited scope of recursive thinking.

### Delayed disposals of nonperforming loans in the 1990s

The asset price bubbles in the stock and real estate markets collapsed at the beginning of the 1990s in Japan. The bursting of an asset bubble is no exception. Nordic countries also experienced similar collapses at the same time as Japan. The exceptional feature of the episode in Japan is that it took more than a decade to finish the cleanup of the nonperforming loans (NPLs) generated by the bursting of the asset bubbles. The Japanese government declared the normalization of bad loans in 2005, almost 15 years after the bubble collapsed.

This delay in the disposal of NPLs was intentionally chosen to avoid banking panic and related financial turmoil due to huge losses on the banks’ side. Financial regulators (the Banking Bureau of the Ministry of Finance) chose to gradually phase the disposal of bad loans, because their scope of attention was narrowly focused on the banking industry. They tried to maximize the “social welfare” that consists of the welfare of the banking community, taking the responses of the firms and people outside of their scope as totally exogenously given, on the implicit (or unconscious) premise, that the outsiders do not change their actions in response to the financial regulators’ actions or inactions.

Given that the agents outside the scope of the policymakers’ attention do not change their actions in response to policy changes, it is obvious that the slower disposals of bad loans are better for the banking industry, as the losses that banks incur every year can be smaller. However, the delay in the cleanup of bad loans means a persistent lingering debt overhang on the borrowers’ side. There are huge and negative side effects of debt overhang on the thinking and actions of agents outside of the banking community.

Here, we point to two side effects of debt overhang: uncertainty and discouraged efforts. The most severe side effects came from uncertainty about the NPLs: who were the borrowers of bad debt, and how much did they borrow? In the 1990s, no databases or official statistics on NPLs were available in Japan. Bank regulators tended to keep the nonperforming loans problem secret within the inner circle of the banking community, and they were reluctant to investigate bad loans and to make the results open to the public. In this environment, the lingering debt overhang created severe uncertainty for firms and consumers during the 1990s. Because nobody knows who the borrowers of bad debt were, a firm or an individual was exposed to huge counterparty risk; that is, the uncertainty of whether the counterparties pay as planned or whether the trading relationships continue as planned, because the counterparties may go bankrupt suddenly if they were the borrowers of bad debt. This is the rise of counterparty risk in the 1990s in the Japanese economy, which resulted in an economic slowdown and shrinkage of supply networks among firms. I call the shrinkage of firms’ networks *debt disorganization* [8, 11]. The shrinkage of the networks of firms implies that the degree of division of labor among firms decreases, leading to lower productivity. The disorganization of supply networks could be the reason for the low productivity and low growth in the 1990s in Japan [9]. Bank regulators could not take account of this huge negative externality in their decision to delay the cleanup of bad loans. They could have noticed the huge negative externality only if they tried harder to think about people outside the banking community.

There is a second externality of debt overhang: an excessively large debt induces a lack of lenders’ credibility, which discourages the borrower from expending effort [12]. It is a feature of debt overhang that [18] pointed out: the borrower hesitates to invest in a new project, because the debt is so large that the borrower expects all returns on the investment to be taken by the lenders’ ex-post (see pp. 29–31 of Sachs [18]). Kobayashi et al. [12] clarify and formalize the idea that this line of thinking emerges, because lenders cannot credibly commit to making repayments smaller than the face value of debt. The inability of lenders to commit causes inefficiency, i.e., inefficiently low effort. Suppose that lenders keep the contractual amount of debt unchanged; nevertheless, they promise to give a sufficient amount to the borrower in exchange for the borrower expending the first-best effort. The lenders’ promise is not trustworthy when the debt is larger than the borrower’s output, because lenders have the legitimate right to take all as repayment. Note that the lender loses credibility only when the debt becomes so large that it exceeds the threshold value. Therefore, debt relief can be effective in restoring the commitment of lenders and reducing the inefficiency caused by their lack of commitment. Policymakers could have noticed the discouragement of the borrower’s effort if they had recursive thinking and imagined how the borrowers think when the debt restructuring of bad loans is delayed.

What is then the optimal policy to avoid the above-mentioned externalities? As we saw above, policymakers may be able to notice the negative externalities of lingering NPLs if they try hard to think about agents outside the banking community. Both uncertainty and insufficient effort were the huge unintentional byproducts of the delay of bad debt cleanup, which policymakers intentionally chose to avoid turmoil in the financial community. The optimal policy was to accelerate the disposal of NPLs and complete the cleanup in the early 1990s. Sweden experienced a bursting of asset price bubbles, which resulted in huge bad loans at the beginning of the 1990s, similar to Japan. Sweden, however, had finished the cleanup of bad loans by 1995 [4], while it took 15 years in Japan.^4^ Swedish economy grew fast in the latter half of the 1990s. Japan could have recovered and grown faster if the cleanup had been completed as early as Sweden.

### Secular stagnation and unconventional monetary policy in the 2000–2010s

Another policy episode in Japan is the unusually long period of unconventional monetary easing. The nominal policy rate in Japan has been zero for almost a quarter century from 1999 to the present, while real economic growth has been stagnant at around 1%, and the inflation rate has been around 0 and only 2% in 2022. The unconventional monetary policy was implemented to induce moderate inflation of around 2% and, eventually, high or moderate economic growth in the long run. The Bank of Japan pioneered the development of new and unconventional monetary policy tools in these years: zero nominal interest rate policy in 1999–2000, quantitative monetary easing policy in 2001–2006, comprehensive monetary easing policy in 2010–2013, quantitative and qualitative monetary easing policy (QQE) in 2013–2016, QQE with a negative interest rate in 2016, and QQE with yield curve control in 2016–present. The BOJ implemented these measures of unconventional monetary policy aimed at dispelling long-standing deflationary expectations in Japan. The arguments behind these policies are basically a quantity theory of money, which states that the price level should increase as the quantity of money increases, given that the velocity of money circulation is (almost) constant (see, for example, [2, 6, 15]). A simple criticism of this argument is that people’s response to monetary policy could change the velocity of money, such that an increase in the quantity of money cannot induce an increase in prices. This is exactly what has happened in the last 2 decades in Japan. On the one hand, these unconventional monetary policies aim to directly change deflationary expectations by changing the BOJ’s commitment to the money-supply schedule. On the other hand, they did not intend to cure the fundamental causes of the low growth that generated deflationary expectations. The fundamental causes of low growth that we discuss below are people’s thinking about the prospect of the economy. Thus, although the BOJ or those who are called “reflationists” were absolutely serious about changing the macroeconomic expectations, they relied on the logic of the quantity theory of money too mechanically and could not change the people’s expectations during the deflationary years of the 2000s and 2010s. There should be something more that policymakers could have done to think about people’s thinking.

We can reinterpret the deflationary expectations as the expectations of low economic growth. Several hypotheses explain how economic growth persistently slows down. Deflationary expectations could be dispelled once the causes of low growth were resolved. Here, we see two of these hypotheses: the first is an increase in income risk, and the second is an increase in the risk of a rare disaster, that is, a government debt crisis.

Krebs [14] theoretically shows that a persistent increase in idiosyncratic income risk lowers the economic growth rate. Krebs [14] notes that the return on human capital is subject to an idiosyncratic labor income shock, because human capital works in the form of labor quality improvement. In a variant of Aiyagari’s [1] model with human capital, Krebs showed that a permanent and exogenous increase in uninsured idiosyncratic labor income risk induces an increase in investment in physical capital and a decrease in investment in human capital. On the one hand, as physical capital is a safe asset in Krebs’ model, investment in physical capital increases, because people increase precautionary savings in response to an increase in labor income risk. On the other hand, investment in human capital decreases, because it becomes a riskier asset as labor income risk increases. Krebs shows that in an economy where human capital plays a crucial role in economic growth, an increase in labor income risk slows down economic growth. As is well known, the number of non-regular workers increased in Japan’s long stagnation since the late 1990s, as the deregulation in that period enabled the firms to employ non-regular workers in various fields. In the 1980s, the ratio of non-regular workers was about 20%, and most of them were housewives who worked part time, while the ratio rose to about 40% in the 2010s. This change indicates a secular increase in labor income risk for Japanese households in recent decades. Thus, we can say that one major factor of deflationary expectations, that is, the expectations of low growth in Japan, could be the secular increase in labor income risk, which cannot be cured by monetary easing.

Another factor is the fear of a rare disaster such as a government debt crisis [12]. Kobayashi and Ueda constructed a simplified version of [7] model of a rare disaster to analyze the effect of the fear of a government debt crisis on the long-term growth rate. Kobayashi and Ueda formalize a government debt crisis as an event in which a large one-time tax increase is imposed on private capital to redeem part of the outstanding government bond. We assume that tax revenue is insufficient and government debt increases in normal times and that a debt crisis, that is, a one-time capital tax imposition, occurs stochastically. It is assumed that the exogenous probability of a government debt crisis is increasing in an outstanding amount of government bonds. In this model, we show that the fear of a debt crisis increases as government debt increases and that an increase in fear slows down economic growth. This is because capital accumulation decelerates as the risk of debt crisis increases. Capital accumulation decelerates, because the capital stock becomes riskier as government debt increases, as people anticipate that a large tax will be imposed on the capital stock in a debt crisis. According to [13], deflationary expectations of low growth are generated by the fear of a government debt crisis. Thus, deflationary expectations can be dispelled in this model only if fiscal reforms restore the sustainability of government debt. This problem cannot be resolved by unconventional monetary easing.

Policymakers could have noticed that idiosyncratic labor income risk and aggregate risk of a rare disaster may have generated deflationary expectations if they thought more seriously about people’ thinking. These examples imply that the optimal policy to restore higher economic growth that enables the BOJ to normalize monetary policy is (i) to reduce income inequality through redistribution and (ii) to restore fiscal sustainability in normal times by increasing less distortive taxes and reducing government expenditures.

### Restrictive PCR testing during the pandemic in 2020–2022

The third policy episode concerns public health measures aimed at the containment of COVD-19 infections in 2020–2021. The unique feature of Japan’s public health policy during this period is the extremely slow increase in the capacity of polymerase chain reaction (PCR) and antigen testing for COVID-19. The number of PCR tests for COVID-19 reached several hundred thousand cases per day in the United States and Western European countries by the summer of 2020, while in Japan, it was only 20 thousand. I argue here that the extremely slow expansion of testing capacity in Japan might have been caused by the narrow scope of recursive thinking on the side of the policymakers in charge of the pandemic countermeasures.

Because pandemic countermeasures such as lockdowns seriously affect economic activities, we can say that the public health policy at this time of the pandemic unintentionally and inevitably works as an economic policy. It is also true for the PCR and antigen testing of this disease. As COVID-19 infection generated a huge number of asymptomatic patients, we suddenly faced uncertainty during the outbreak. The uncertainty is that we cannot know whether our trading partners or ourselves were infected. This is a sudden emergence of large and unprecedented uncertainty in the economy, which discourages people from expanding transactions with non-regular customers because of the fear of infections. Thus, the uncertainty of infection exerts a huge negative externality that excessively discourages economic activity; therefore, the reduction of this uncertainty is a legitimate goal of economic policy. PCR and antigen tests are highly effective measures to reduce the uncertainty of infections, even if the possibility of false positives or false negatives is discounted. From the viewpoint of economic policy, it is reasonable to seek an increase in the capacity for infection testing at a maximum speed.

One factor that realized the rapid expansion of the PCR testing capacity in the US or European countries could be that the policy objectives for the pandemic countermeasures were set successfully to include the reduction of uncertainty in the economic system, not just to contain infections and provide medical treatment to the patients appropriately.

In the early stage of the pandemic, there was a clear consensus among the majority of the people in various fields in Japan that the capacity of PCR testing should be increased as fast as possible to reduce the uncertainty of infection in society. One such example is the policy proposal submitted by 114 prominent leaders in various fields in Japan to Yasutoshi Nishimura, the minister in charge of the pandemic countermeasures at that time in June 2020. This policy proposal, which I was involved in, strongly demanded the government increase its testing capacity to reduce uncertainty and normalize socio-economic activities in Japan.

See the following URL for the proposal (written in Japanese): https://www.rieti.go.jp/users/kobayashi-keiichiro/covid-19-proposal.pdf.

See the list of 114 proponents of the proposal ( in Japanese): https://www.rieti.go.jp/users/kobayashi-keiichiro/supporters.pdf.

However, the expansion of testing capacity in Japan was extremely slow, and the number of cases per capita was around one-tenth of the other major countries by 2020. In my understanding, this is because of the jurisdictional or disciplinary divide of public health policy or, in our terminology, the limited scope of recursive thinking on the side of public health experts in the government. Public health experts, most of whom are medical doctors, consider that the sole purpose of the test is to efficiently find infected persons and bring them to appropriate medical treatments. They do not think that the reduction of uncertainty in economic activity is the purpose of PCR testing for COVID-19. Although they may understand the logical causality that the PCR test reduces market uncertainty, they cannot think that such an effect is important as a function of the PCR test. They thought that the reduction in uncertainty might be just a theoretical possibility, which is an unintended byproduct of testing. Since policymakers believe that pursuing efficiency in finding infected people is their mandate, they thought that testing many people at random to find many test negative is just a waste of time and resources, because testing negative means that the test failed to find an infected person. Failing to find an infected person means the failure to attain the purpose of testing for public health policymakers. Therefore, the natural conclusion for Japanese policymakers is to restrict the objects of PCR testing to people with a high probability of infection, such as those in close contact with patients. To achieve efficiency in the use of time and resources, it may also be optimal to restrict the total number of testing cases.

All these arguments of medical experts are overturned from the viewpoint of economic policy. The PCR and antigen test of COVID-19 work as an economic policy, as it can reduce the uncertainty of infection that workers and customers individually face in the marketplace. Reduction in uncertainty enhances economic transactions and improves output and social welfare. From the viewpoint of economic policy, policymakers should reduce uncertainty by increasing the number of tests as much as possible and making anyone who wants to be tested able to be tested at any time. Testing negative is not wasting time or resources, but it provides very useful information that reduces the uncertainty of infection. It is not a failure but a success for economic policymakers, as it provides useful information that you are not infected (with a 70% probability).

The most serious problem for pandemic countermeasures in Japan is that these viewpoints of public health and economic policy were not integrated, and the decision on the expansion of testing capacity is dominated by the views of medical policy only. If medical policymakers imagine more seriously the thinking of people who are outside their jurisdiction, their policy decisions may have changed.

Social welfare, which consists of welfare from economic activities and the reduction of infections, should have been maximized by accelerating the expansion of testing capacity in 2020. The decrease in the GDP growth rate during 2020 was almost the same in Japan as in the US and EU, even though the number of confirmed patients per capita is one-tenth in Japan compared to the US or EU. This fact implies that there is a possibility that economic activities in Japan were depressed excessively due to the huge uncertainty of infections that were widespread, because people could not be tested when they wanted in their everyday life during 2020 and 2021.

## Why does policymakers’ lack of recursive thinking emerge?

The restricted scope of recursive thinking, or the disciplinary (or jurisdictional) divide, has long been formed. It is fixed as a framework of thought in the mind of policymakers through education and experience over a long period. Therefore, the scope cannot be changed sufficiently quickly in a time of crisis, so that the optimal policy response to the crisis can be chosen in a timely manner. Having said that, there should be some problems unique to Japan, as the above-mentioned policy episodes show differences in the policy responses of Japan from those of other countries. There may be organizational factors or irrational prejudices that strengthen the limited scope of recursive thinking.

An example of such an organizational factor is Silo mentality (or Sectionalism) in government agencies or the community of experts. Once a community of professionals or experts is formed, communication between the inside and outside of the community tends to weaken. This tendency may be conspicuous in Japanese government agencies.

A simple and irrational prejudice held by policymakers, which may be fostered by Silo mentality, is the (unconscious) premise that people are ignorant and do not have the ability to think. It is an utter denial of the critical attribute of the humanity of people.

This Silo mentality and the prejudice that people are ignorant, which is sometimes observed in policymakers’ mentality, may have a historical origin in bureaucracy since the feudal age. In any case, life is easy for policymakers if they simply hold the view that people do not have the ability to think, because then policymakers do not have to think about their thinking.

This totally unacceptable view of human nature survived, because the policymakers’ communities were closed, and scrutiny from outside was rarely conducted. Moreover, the communities of bureaucrats or experts were recognized by the majority of people as unchallengeable authorities in their respective fields. Government agencies need to be reformed to be more open, and citizens themselves should have a more critical view of the government’s or experts’ authority. The reforms of the Silo mentality in the government agencies have been attempted for decades unsuccessfully, e.g., Third Provisional Commission for Administrative Reform. It may be helpful to proceed the government reforms to break the Silo mentality if we can provide a commitment device for the government. One example of the commitment device for fiscal policy is to establish an independent fiscal institution (IFI) that provides an intergenerational forecast of fiscal sustainability as a neutral body that is independent of the government.^5^

## Conclusion

In this study, we observed that policy failures in the last several decades in Japan were related to the lack of or limited scope of recursive thinking on the side of policymakers. A crucial lesson from these policy episodes is the necessity to broaden the scope of recursive thinking in the mindset of policymakers. This goal should be widely shared in the arguments for the structural reforms of regulations and government agencies.

Finally, I argue two examples of policy agendas that seem quite difficult to resolve under the existing framework of thought or the given scope of recursive thinking. The first one is the nuclear deterrence strategy [10]. We usually consider nuclear deterrence a two-party game between conflicting nuclear powers, and we neglect the rest of the world (ROW). We usually do not care about how the ROW thinks and responds to the nuclear escalation. The Ukrainian war has brought the use of nuclear weapons into the realm of possibility. The ROW will incur unbearable costs if a full-scale nuclear war breaks out between the two nuclear nations. If we consider the thinking and response of the ROW, we can consider the following international mechanism to prevent the escalation of the nuclear conflict. As a mechanism to prevent some parties from ascending the first step of a nuclear ladder, it would be beneficial to create a treaty that imposed sanctions for the preemptive use of nuclear weapons, to which many nations (i.e., the ROW) would become parties, that would “impose immediate, unconditional, and maximum sanctions against any nation that launched a nuclear preemptive strike.” If a treaty on the sanctions for the preemptive use of nuclear weapons can be created, sanctions can be implemented even if the signatories are non-nuclear weapons states, so the creation of this treaty would create a new international norm with high credibility. Creating the new treaty is essentially tantamount to the creation of an international norm of equity that all citizens in various countries in the world should share the power to decide the fate of this planet equally.

The second example is decision-making regarding intergenerational problems, such as the long-term sustainability of government debt and global warming. The intergenerational problem is an investment project in which the present generation must pay the cost of investment, while only the future generation obtains the return on the investment. Since the return accrues to the generation, which is different from those who pay the cost, rational and selfish people tend to push off the intergenerational problem to the next generation until it becomes too late. Any political decision-making system, either democracy or autocracy, is powerless to solve this intergenerational problem, because decision-makers are all in the present generation. The most urgent agenda is to let the present generation act to restore intergenerational sustainability in various policy fields. In other words, it makes the present generation more altruistic toward future generations. One endeavor to make the present generation more altruistic is future design (FD), which is a research program to change the decision-making process of the present generation by introducing a role-playing game in which participants play the role of the future generation (see Saijo [19] for more details of the FD). In the FD experiments, a group of people discussed the policy problem of the present time from the viewpoint of the people 50 years in the future. One example is a discussion about the water supply in Yahaba town, Iwate prefecture, held in 2015 [20]. As the water supply earned a surplus at that time, most of the residents wanted to cut the price to share the surplus among them, the present generation. The town office organized a discussion of the residents, in which groups of five or six people were formed, and they were asked to think as if they were Yahaba residents in 2060 and consider the water supply from 2015 to 2060. In this experiment, the people who played the role of the 2060 residents noticed the necessity for a large renewal investment in water supply facilities and water pipes in the period between 2015 and 2060, and concluded that they should raise prices and accumulate funds for the future. Based on this discussion, the town office raised the price of the water supply in 2017. FD is a project that expands the scope of recursive thinking for future generations. Recursive thinking is impossible with the future generation, as there is no loop between the present generation and the future generation thinking about each other’s thinking, because the future generation is nonexistent yet. The FD is trying to form an “imaginary” recursive thinking with the “imaginary” future generation, where the imaginary future generation is the people (of the present generation) who play the role of the future generation.

These are two examples of our new policy challenges. A key is to expand the scope of our thought about thinking of others, which includes non-nuclear nations in the game of nuclear deterrence and imaginary future generations in sustainability problems. Introducing recursive expectations into policy analysis has been the most significant contribution of economics since the adoption of the rational expectations theory. Further progress should be made on this contribution in response to new policy challenges.

## Data Availability

I have nothing to state, as I do not use any data in this paper.

## Footnotes

1 In the recursive expectations equilibrium, the expectations are the fixed point of the expectation operator that maps the expectations on the allocation, which is then mapped to the new expectations.

2 Although [16] argues based on the rational expectations hypothesis, the point is that the recursive thinking, not the perfect rationality, in the two components of the rational expectations is crucial in the Lucas critique.

3 This is true not only for government officials but also for all individuals. As everyone should notice the importance of recursive thinking, we should teach recursive thinking systematically in schools and institutes for education.

4 Sweden finished the cleanup of the NPLs very rapidly, because it was under pressure of reforms from the foreign investors, whereas Japan was not exposed to the foreign pressures, because the Japanese economy was a world-largest lender to foreign countries.

5 I thank Tomomi Miyazaki for raising this point. I also thank an anonymous referee for pointing out that commitment devices are needed to mitigate the negative effects caused by the lack of recursive thinking if the Silo mentality cannot be altered in Japan.
